# Tuberculosis treatment delay and contributing factors within tuberculosis patients in Ethiopia: A systematic review and meta-analysis

**DOI:** 10.1016/j.heliyon.2024.e28699

**Published:** 2024-03-23

**Authors:** Getahun Fetensa, Dessalegn Wirtu, Belachew Etana, Bizuneh Wakuma, Tadesse Tolossa, Jilcha Gugsa, Dabesa Gobena, Ginenus Fekadu, Misganu Teshoma Ragasa, Eshetu Ejeta

**Affiliations:** a Department of Nursing, School of Nursing and Midwifery, Institute of Health Sciences, Wollega University, Nekemte, Ethiopia; b Department of Public Health, Institute of Health Sciences, Wollega University, Nekemte, Ethiopia; c Oromia Physician Association, Addis Ababa, Ethiopia; d Batu General Hospital, Oromia Regional Health Bureau, Addis Ababa, Ethiopia; e Department of Health, Behavior, and Society, Faculty of Public Health, Institute of Health, Jimma University, Jimma, Ethiopia; f Public Health Emergency Management and Health Research Directorate, Oromia Health Bureau, Addis Ababa, Ethiopia; g School of Medical Laboratory Science, Institute of Health, Jimma University, Jimma, Ethiopia; h Department of Pharmacy, Institute of Health Sciences, Wollega University, Nekemte, Ethiopia; i Department of Midwifery, School of Nursing and Midwifery, Institute of Health Sciences, Wollega University, Nekemte, Ethiopia; j Department of Public Health, Ambo University, Ambo, Ethiopia; k Deakin Health Economics, School of Health and Social Development, Institute for Health Transformation, Deakin University, Geelong, VIC, Australia

**Keywords:** Tuberculosis, Delay in treatment, Patients, Ethiopia

## Abstract

**Background:**

Tuberculosis (TB) is a significant public health disease and a major contributor to illness and death worldwide, including in Ethiopia. There are many information from first source which had inconclusive result in Ethiopia. Therefore, this review aimed to produce pooled evidence on the TB treatment delay and factors associated with it.

**Methods:**

The absence of a similar study with a systematic review and meta-analysis was confirmed. Articles from online available and unpublished sources conducted within Ethiopia between 2002 and 2024, were thoroughly screened using electronic sources such as Medline, Embase, Hinari, PubMed, the Cochrane Library, the Web of Science, and Google Scholar. Data analysis was performed using STATA version 14. Heterogeneity was assessed using Inverse of Variance (I^2^) and Cochrane Q tests. The funnel plot was employed to rule existence of publications subjectively while bias was checked using Egger's statistical method to quantify the bias.

**Result:**

Prevalence of TB treatment delay in Ethiopia was 50.42% at 95% (43.21, 57.64). Factors such as knowledge about TB, distance to health facilities less than 10 km, initial contact at a government service providing center for TB, having some educations, having pulmonary Tuberculosis, urban residency, were prtotective towards treatment delay. Female in gender, no chest pain symptom, disease severity with no restriction on daily activity, alcohol drinkers, and unmarried respondents were at higher risk to miss on time tuberculosis treatment.

**Conclusion and recommendation:**

The tuberculosis treatment delay in Ethiopia was considerably unexpected and basic personal variables and facility related variables were statistically associated with treatment. Therefore, Ethiopian TB control programs have to recognize and tackle the problem, obstacles, and vulnerability across the continuum patient care taking down and connecting to treatment post-diagnosis. This can be achieved by capacitating both government and non-governmental service provision centers and minimizing unfilled difference across professional awareness and skill, which will contribute further to minimizing delay.

## 1 Introduction

### 1.1 Background

Tuberculosis (TB) is not only a public health-important disease but also it is a high contributor to illness and death throughout the world including in Ethiopia [[1], [2], [3], [4], [5]]. Predictions indicates one fourth of the global communities are attacked with the tuberculin, with a life time risk ranging from 5 to 10% of progression into TB disease [6]. Because of its high level of infectiousness [7]. Coordinating, individual patient as individual as well as equal, and thwarting at early and availing TB services for all nation, considering all forms of tuberculosis was also stated as one of the Sustainable Development Goals Goal 3.3 [4].

Worldwide, there is a large increase in new TB cases from 5.7 to 5.8 million were diagnosed for TB per year during 2009–2012(8). World Health Organization (WHO) predicted that around 54 million TB deaths were halted during between 2000 and 2017 due to strengthened disease aversion and treatment, and provision of care; however, up to 10 million person will be diagnosed with tuberculosis annually [3,9,10]. Non-on time in tuberculosis (TB) treatment is the leading hider to the appropriate treatment of the diseases [11]. Immediate intervention is needed to modify accessibility and standard of care. The WHO attempted various efforts to scale down TB screening and treatment in 2018 using a plan to meet prediction that a minimum of 30 million people will be candidate for tuberculosis eradication management during 2018 and 2022 [12]. There is also a national End TB policy with a target to eradicate the TB un usually increase of tuberculosis infection by decreasing TB-associated deaths by 95% and by stopping incidence of TB cases with 90% during time of 2015 and 2035.

The number of deaths and the incidence of Tuberculosis (TB) has steadily declined since 2000, falling by 20% between 2000 and 2015 [13]. As per to the 2007, World Health Organization (WHO)predictions, new tuberculosis infection of any type and smear-positive tuberculosis stop at 341 and 152 per 100,000 population, chronologically in Ethiopia [2]. The issue was contributed by factors constituting limited resources for tuberculosis such as a limited numbers of health professionals, limited important facilities, and limited TB screening materials and re-agents [13]. Furthermore, Ethiopia arrived the half of the goal in decreasing of majority of the Millennium Development Goals (MDGs) goals associated with the program. But, the decrement of TB new and existing TB cases rates remained relatively minimum [14].

Although medications for TB are without cost for any patient as it comes from different funders, the disease have potential considerable financial burden which can be a reason for treatment delay [7]. Ethiopia is among countries burdened by the problem [2,8]. As per information from the Ethiopian Ministry of Health hospital statistics data, TB is the leading cause of death, the third reason for hospitalization (following deliveries and malaria), and the second contributor to mortality in Ethiopia, following malaria [2]. Huge number of care seekers, loose TB laboratory samples referral methods, and inadequate of TB identification materials are the contributors for the delay and consequences of the delay. There also evidence of low TB the treatment success rate and cure were with significant concern as it was 79.4%, and 18.3% respectively [15]. There is report of unsuccessful treatment rate 5.6%, in which 9 (0.3%) un succussed treatment, 2.5% died, and 2.7% were with draw from treatment 18).

Ethiopia have estimated per year decrement of TB new cases greater than 16%. The country agrees well bringing cases to zero the TB epidemic prior to 2035 and directing at TB eradication in the foreseeable future [16]. Beside this, on time case detection and effective management of cases with tuberculosis can halt millions of mortalities every year. However, there might still be persistent gaps in case detection and treatment. This can misdirect policymakers and practitioners in controlling TB [3,10]. Furthermore, fear of stigma toward tuberculosis will contributes to delays in treatment-seeking [17,18]. Delay only within one month contributes to 90% of lung degradation [19,20]. Unwillingness or poor social support for patients with TB from others contributes to increasing economic impact on them [7].

It embraced that knowledge and perception of patients and their family members leading to an unreplaceable role in treatment [18]. The stigma related to TB may lead to non-on time visit of health facility, bad drug taking habit, and inappropriate prognosis [7]. The number of TB treatment delay in Ethiopia ranges from 3.03% to 79.84% [21,22]. Although there were plenty of primary studies on tuberculosis treatment delay in Ethiopia, they came up with inconsistent and inconclusive findings that indicate a clear need for pooled evidence. As far as our knowledge is concerned, this kind of study is the first of its kind in Ethiopia with the same topic and design. Hence, the results of the current analysis will be helpful for policymakers, health service program managers, and the scientific community. Therefore, this study aimed to identify the overall prevalence of TB treatment delay and contributing factors in Ethiopia.

## 2 Methods and materials

### 2.1 Search strategy

To avoid duplication, we checked for similar systematic reviews and meta-analyses on this topic. We searched for online available and non-online studies from 2002 to April 1, 2024, using various electronic sources, such as Medline, Embase, Hinari, PubMed, the Cochrane Library, the Web of Science, and Google Scholar. We also searched the electronic libraries of Ethiopian universities for unpublished papers. We used pre-determined search engines and Medical Subject Headings (MeSH terms) to find all the relevant articles conducted in Ethiopia. We adapted the search engine for different electronic databases with aid of appropriate conjunctions/connectors and important terms like "(((((prevalence, TB treatment/diagnosis delay) OR magnitude, TB treatment/diagnosis delay) AND associated factors, TB treatment/diagnosis delay) OR determinants, TB treatment/diagnosis delay) AND TB treatment/diagnosis delay) AND Ethiopia *with first keywords "(((prevalence, TB treatment/diagnosis delay) OR magnitude, TB treatment/diagnosis delay) AND associated factors, TB treatment/diagnosis delay) OR determinants, TB treatment/diagnosis delay) AND TB treatment/diagnosis delay) AND Ethiopia.”* The overall analysis was embraced using the Preferred Reporting Items for Systematic Reviews and Meta-Analyses (PRISMA) guideline [23]. Endnote (version X7.2) applied to handle downloaded literature for duplicate and manage citations as well as ease the process of articles evaluation.

### 2.2 Selection and eligibility criteria

**Fig. 1:**
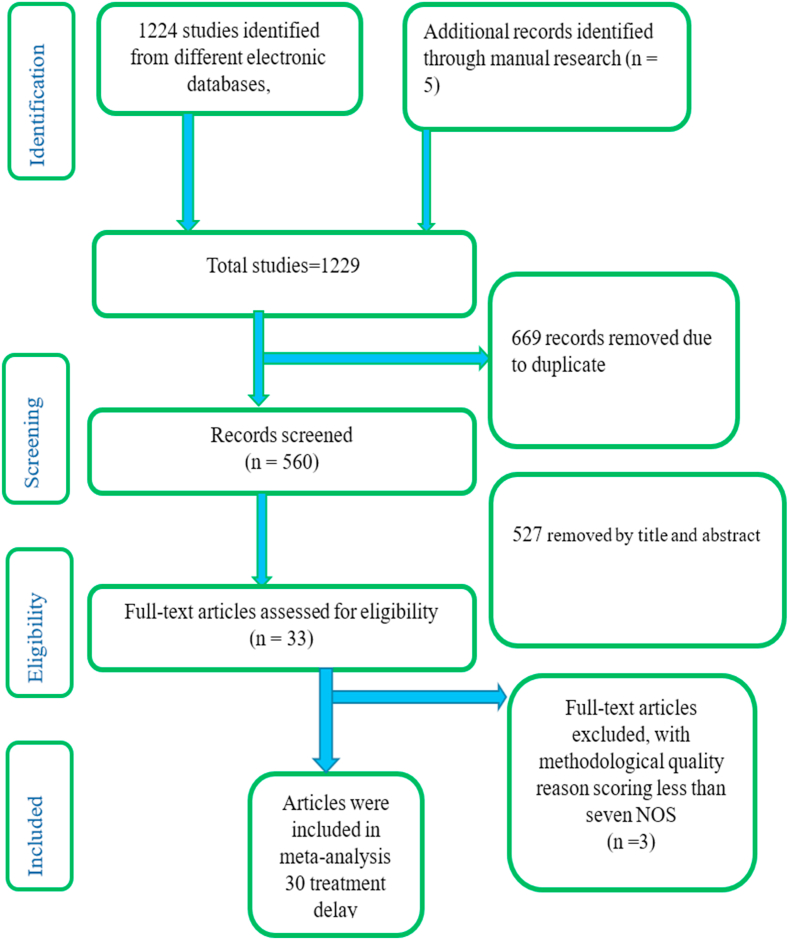
PRISMA flow diagram of included studies in the systematic review and meta-analysis of tuberculosis treatment delay in Ethiopia, 2024.

Any study which includes cross-sectional, case-control and cohort study designs that consisting the delayed treatment and contributing variables of tuberculosis were included. Articles were limited to the English language. The study only included quantitative studies, as qualitative studies could not provide pooled evidence for the figure. It also excluded studies with multi-drug-resistant TB. The authors reported the sample size of the primary studies. Results with a Newcastle-Ottawa Scale (NOS) score of less than 7 were excluded. The review utilized the CoCoPop (condition, context, and population) criteria to screen studies for inclusion or exclusion. The condition (Co) was the magnitude of treatment delay, the context (Co) was only studied in Ethiopia, and the study population (Pop) was patients diagnosed with tuberculosis. The overall search yielded 1224 articles, of which 669 were removed due to duplicates and 560 were screened for eligibility. From the 560 articles screened for eligibility, 527 were removed by their titles and abstracts. A total of 33 articles were assessed for full text, of which three articles excluded due to their NOS less than seven (Fig. 1).

### 2.3 Measurement of the outcome

The current analysis addressed two outcomes. To measure tuberculosis treatment delay, which was the percentage of TB patients who started drugs later than recommended one of the outcomes. The second was to determine the variables contributing to treatment delay within TB clients, which were reported as odds ratios focusing on the two outcomes from the original results. Delay in treatment is the time ranges from TB identification and initiation of *anti*-TB medications [8,24].

### 2.4 Quality screening and data extraction

We used Endnote version X7.2 to manage the data and remove duplicate articles. Quality screening of each paper using the Newcastle-Ottawa Scale (NOS), which is suitable for observational studies. Two authors, GF and TT, independently taking the information with assist of a standardized template on Excel spread sheet. The template included the author/s name, publication year, region, study design, sample size, and number of cases for the first objective. Associated variables were extracted the data as two-by-two tables and calculated the log odds ratio for every variable from the primary studies. We resolved any discrepancies between the two authors by discussion.

### 2.5 Statistical analysis and synthesis

We conducted a systematic review to describe and compare the primary studies. We used meta-analysis for the analysis, using STATA version 14 software. We used the “generate” command in STATA to obtain the log and standard error of the odds ratio (OR) for individual selected article.

### 2.6 Assessment of heterogeneity

The funnel plot, the Q test, and the inverse variance index were used to visually analyze heterogeneity (I^2^). Additionally, the Egger statistical test and the I-squared statistic (I^2^ = 100% (Q-df)/Q) were employed to assess the presence of publication bias. A P-value ≤0.10 deemed associated for the Q test, signifying pronounced study heterogeneity. Based on the outcome of I^2^, the model was chosen for analysis. As a result, to quantify the aggregate effect of patient treatment delays, we employed a random effect model for outcomes with I^2^ > 70. We looked at a potential source of heterogeneity. An analysis based on the area where main research was conducted was used to try to investigate the source.

### 2.7 Ethical issue

Not relevant. Not applicable for current study directly. However, all primary studies included within current review were ethically approved and the research adhered to research ethical principle. As well evidences were found from online data bases.

## 3 Result sociodemographic

### 3.1 Characteristics of included studies for the prevalence of delay in treatment of TB

A total of 12,352 research participants from Oromia, Amhara, SNNP, Afar, Harar National Regional State, and Addis Ababa city were included in 30 studies from ten regional states and one municipal administration. Accordingly, nine studies were from Amhara regional state, four from SNNP, one from Afar, one from Harar, one from Sidamma [25], one Tigray [26], Eight from Oromia, and one Addis Ababa city administration [27] (Table 1).

**Table 1:** Characteristics of selected articles in determining the pooled prevalence of treatment delay among TB patients in Ethiopia, 2024.

S.n	Author	Year of publication	Study design	Region	Area	sample size	NOS
1	Solomon Yimer et al. [29]	2005	Cross-sectional	Amhara	Zones of Amhara	384	9
2	Tatek Wondimu et al. [21]	2007	Cross-sectional	Oromia	East wollega	198	8
3	Solomon Y et al. [30]	2011	Cross-sectional	Amhara	Zones of Amhara	211	10
4	Awol Hussen et al. [31]	2012	Cross-sectional	Oromia	Bale zone	129	8
5	Mulugeta Belay et al. [32]	2012	Cross-sectional	Afar	Dubti hospital, Asayta Health center	216	8
6	Anteneh Asefa et al. [33]	2014	Cross-sectional	SNNP	Hawasa area	306	9
7	Zerihun Zerdo et al. [34]	2014	Cross-sectional	SNNP	Gamo Goffa	218	7
8	Yeshiwork A et al. [35]	2014	Cross-sectional	Amhara	Bahirdar	315	7
9	Endalew G et al. [36]	2014	Cross-sectional	Amhara	Bahirdar	360	
10	Solomon A. et al. [37]	2015	Cross-sectional	Amhara	Amhara	240	10
11	Senedu Bekele et al. [38]	2016	Cross-sectional	Amhara	West Gojjam Zone	706	10
12	Dejen Ts et al. [39]	2016	Cross-sectional	Amhara	Wollo	528	10
13	Getinet Shewaseged, et a [27].	2017	Cross-sectional	Addis Ababa	Addis Ababa	425	10
14	Workineh Bekene et al. [40]	2017	Cross-sectional	Harar	Harar Twon	280	7
15	Robel Yirgu et al. [41]	2017	Cross-sectional	Oromia	Arsi Zone	716	9
16	Raghavendra Yarlagadda et al. [42]	2018	Cross-sectional	Oromia	Jimma Zone	105	7
17	Abdurahaman Seid et al. [43]	2018	Cross-sectional	Amhara	Dessie area	382	10
18	Dame Tesfaye et al. [44]	2018	Case-control	Oromia	Mettu town	87	7
19	Abyot Asres et al. [45]	2019	Cross-sectional	SNNP	SNNP	735	9
20	Tirusew Maru et al. [46]	2019	Cross-sectional	Oromia	Adama Town	598	10
21	Gedeyon G et al. [47]	2019	Cross-sectional	SNNP	Hadiya zone	340	7
22	Nsenate A et al. [48]	2019	Cross-sectional	South Ethiopia	Gedeo zone	411	9
23	Kiros T et al. [26]	2020	Cross-sectional	Tigray	Tigray	875	10
24	Muhammed A et al. [49]	2020	Cross-sectional	Oromia	Oromia Special zone	387	10
25	Daniel G et al. [50]	2020	Cross-sectional	Ethiopia	Nationwide	844	10
26	Yibeltal E et al. [51]	2020	Cross-sectional	Amhara	Debremarkos	300	8
27	Asrat A et al. [52]	2022	Cross-sectional	South Ethiopia	Gamo Zone	255	9
28	Amanuel F et al. [25]	2022	Cross-sectional	Siddamma	Hawasa city	196	7
29	Berhane M et al. [53]	2023	Cross-sectional	Oromia	Jimma Zone	1161	10
30	Fentabil G et al. [28]	2020	Cross-sectional	Somali	Somali zones	444	10

### 3.2 The magnitude of TB treatment delay in Ethiopia

**Fig. 2:**
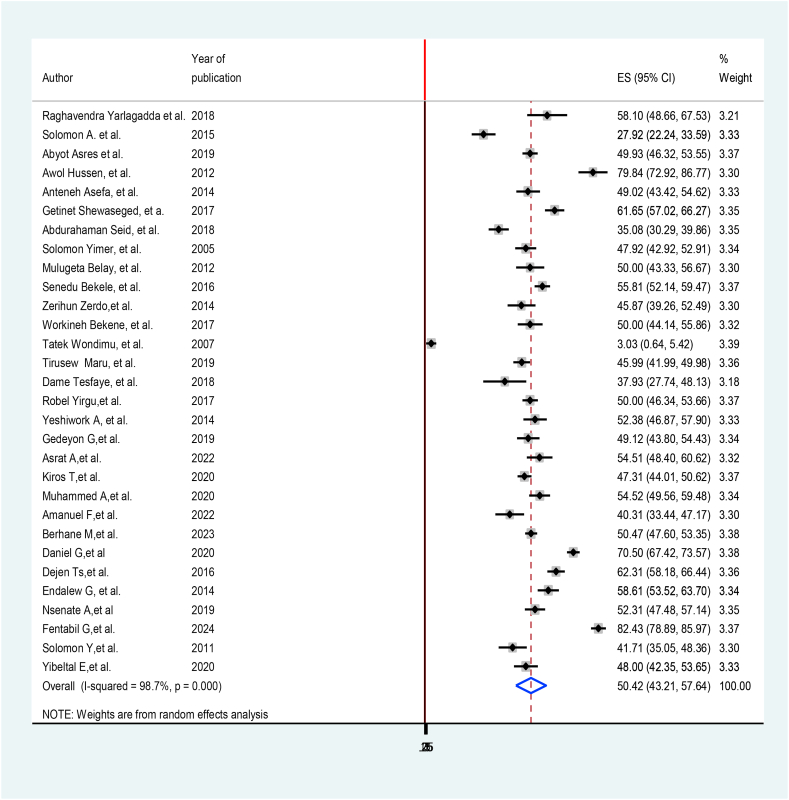
Forest plot of Pooled prevalence of treatment delay among TB patients in Ethiopia, 2024.

The overall prevalence of treatment delay of TB in Ethiopia was 50.42% at 95% (43.21%, 57.64%). The highest magnitude was reported from Somali region 82.43% (78.89, 85.97) [28], while the minimum magnitude was again in Oromia Regional state 3.03% (0.64,5.42) [21] (Fig. 2).

### 3.3 Subgroup analysis

**Fig. 3:**
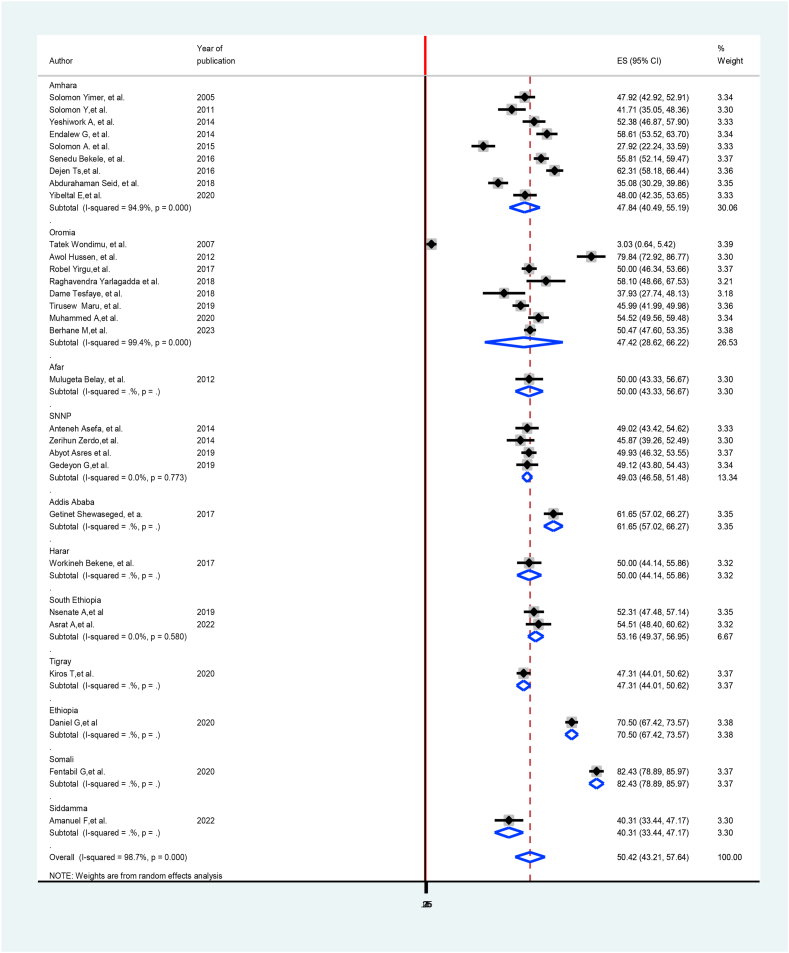
Forest plot subgroup analysis of prevalence of treatment delay among TB patients in Ethiopia: Systematic review and Meta-analysis,2024.

Region within with original studies were conducted were used to undertake sub-group analysis. The minimum prevalence of TB treatment delay was observed in the Siddamma region, 40.31% (33.44%, 47.17%) [25], and the highest in Addis Ababa was 82.43% (78.89%, 85.97%) [28] (Fig. 3).

### 3.4 Publication bias

**Fig. 4:**
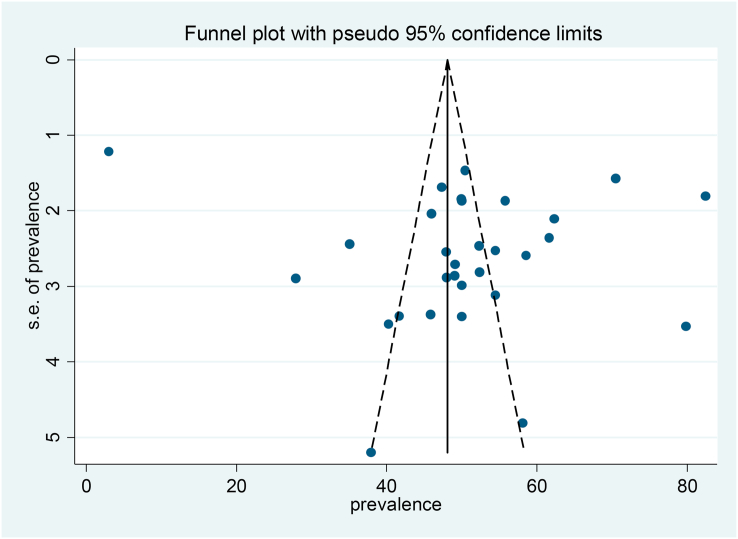
Funnel plot with 95% confidence limit of the pooled magnitude of treatment delay among tuberculosis patients in Ethiopia: Systematic review and Meta-analysis,2024.

Publication bias was assessed subjectively with visual inspection of funnel plot and Egger test to quantify the bias. The result reveal that funnel plot was asymmetrical, and the Egger test indicated presence of publication bias statistically (Fig. 4).

### 3.5 Determinants of treatment delay among tuberculosis patients in Ethiopia

**Fig. 5:**
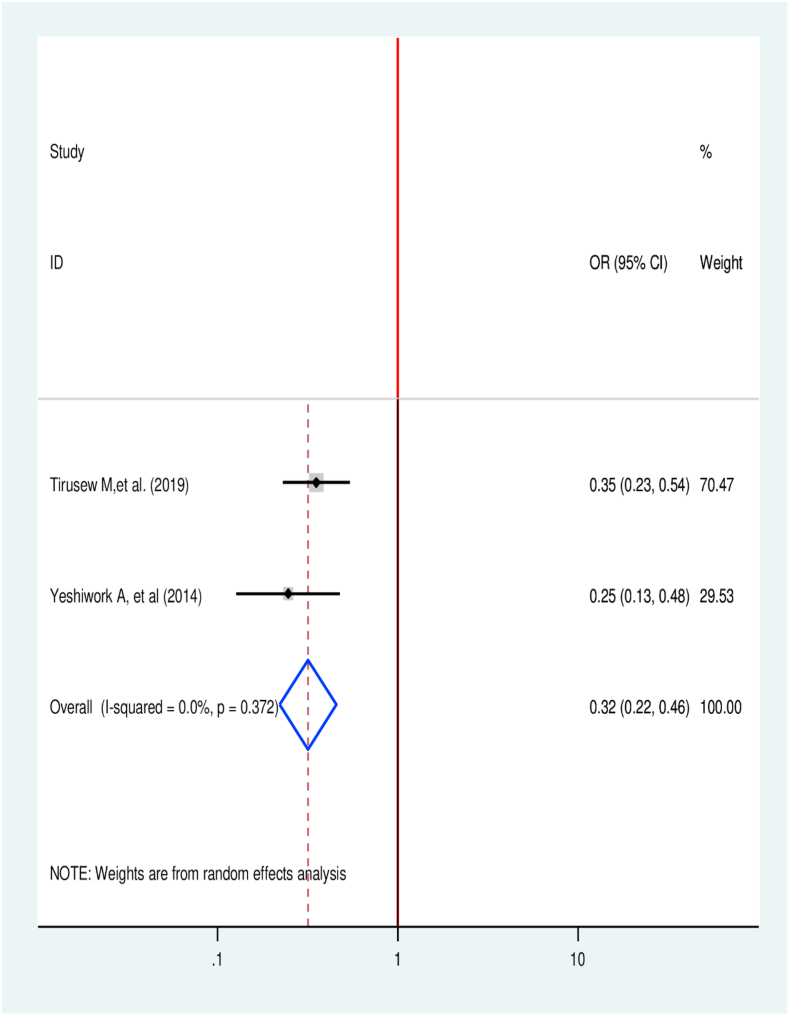
Association between knowledge about TB and treatment delay in Ethiopia: Systematic review and Meta-analysis,2024.

Knowledgeable patients about TB had 68% lower likelihood to delay TB treatment in relation those have poor knowledge (OR = 32, 95% CI 0.22, 0.46) [35,46] (Fig. 5).

**Fig. 6:**
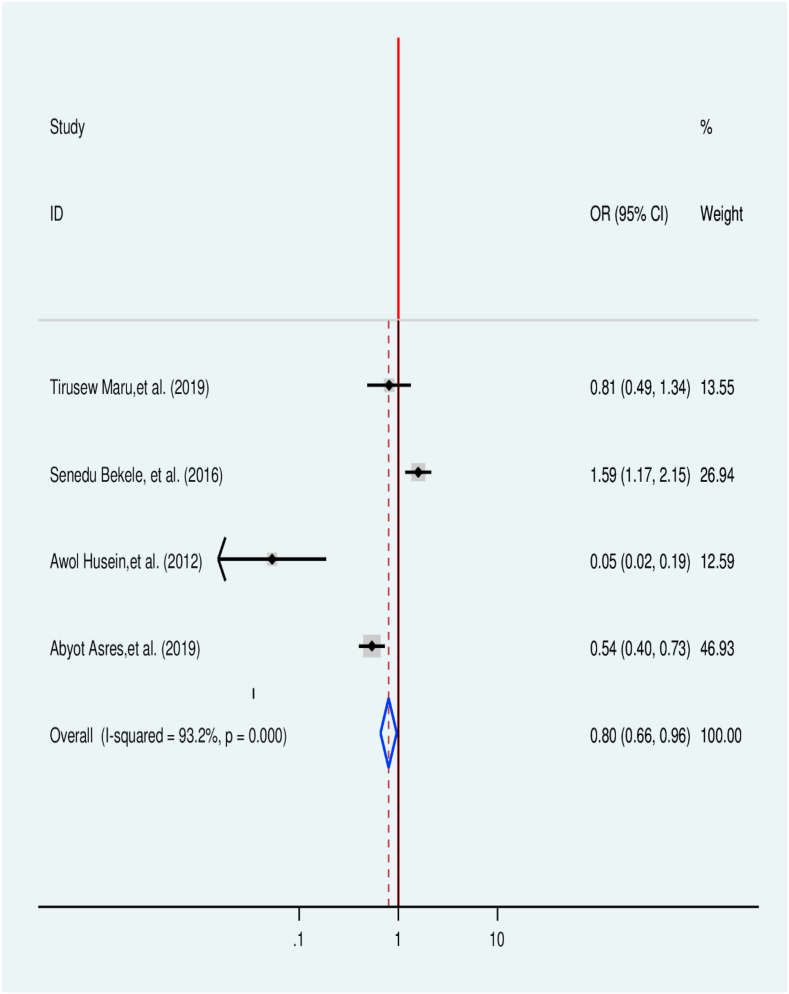
Association between first visited health facility and treatment delay among TB patients in Ethiopia: Systematic review and Meta-analysis,2024.

Four studies containing primary visited health facilities for treatment were screened to evaluate the association between treatment delay for TB and sought health facilities [31,38,45,46]. One of the included studies doesn't have an association with treatment delay, while three of the study have a significant association with treatment delay. The pooled finding indicates that patients seeking care first from governmental health services were 20% less likely (OR = 0.80, 95% CI (0.66, 0.96)) to have treatment delay when compared with those seeking care first from private health facilities. High heterogeneity was observed as studies were from different regions of the country (Fig. 6).

**Fig. 7:**
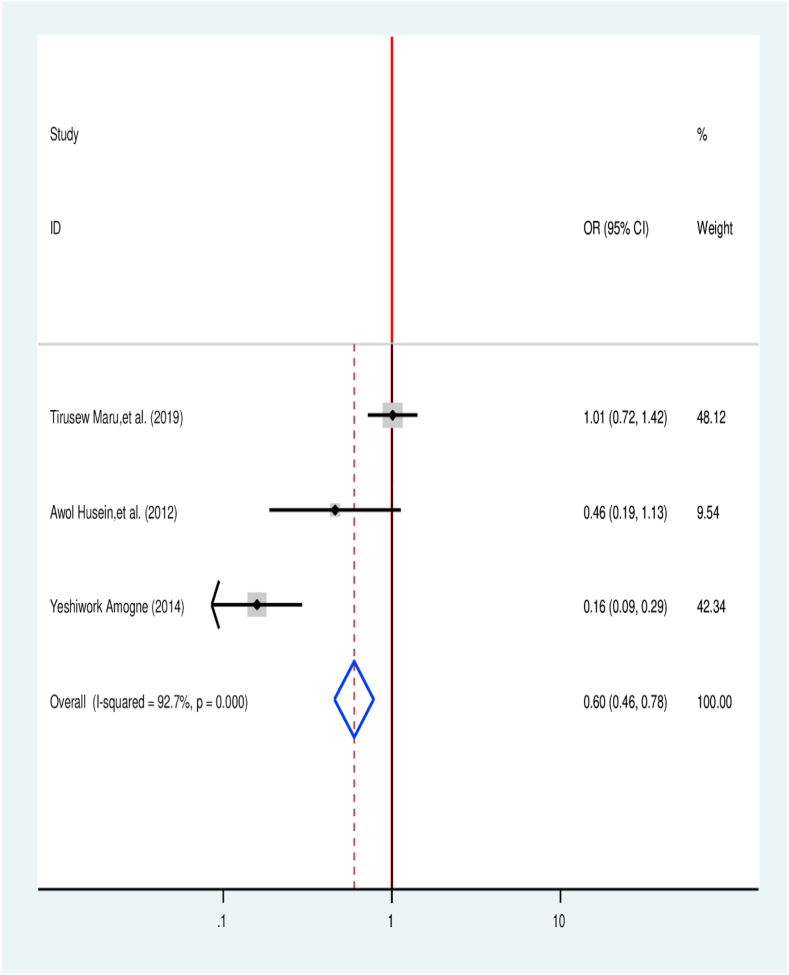
Association between distance from health facility and treatment delay in Ethiopia: Systematic review and Meta-analysis,2024.

The analysis included three studies to identify the association between TB treatment delay and distance from a health facility [31,35,46]. Two of the selected articles have no significant association with TB treatment delay. Among selected one of the articles was statistically associated. Overall effect reveals that patients coming to health facilities from a distance of less than or equal to 10 KM were 40% less likely to delay treatment (OR = 0.60, 95% CI 0.46, 0.78) in comparison with their counterparts (Fig. 7).

**Fig. 8:**
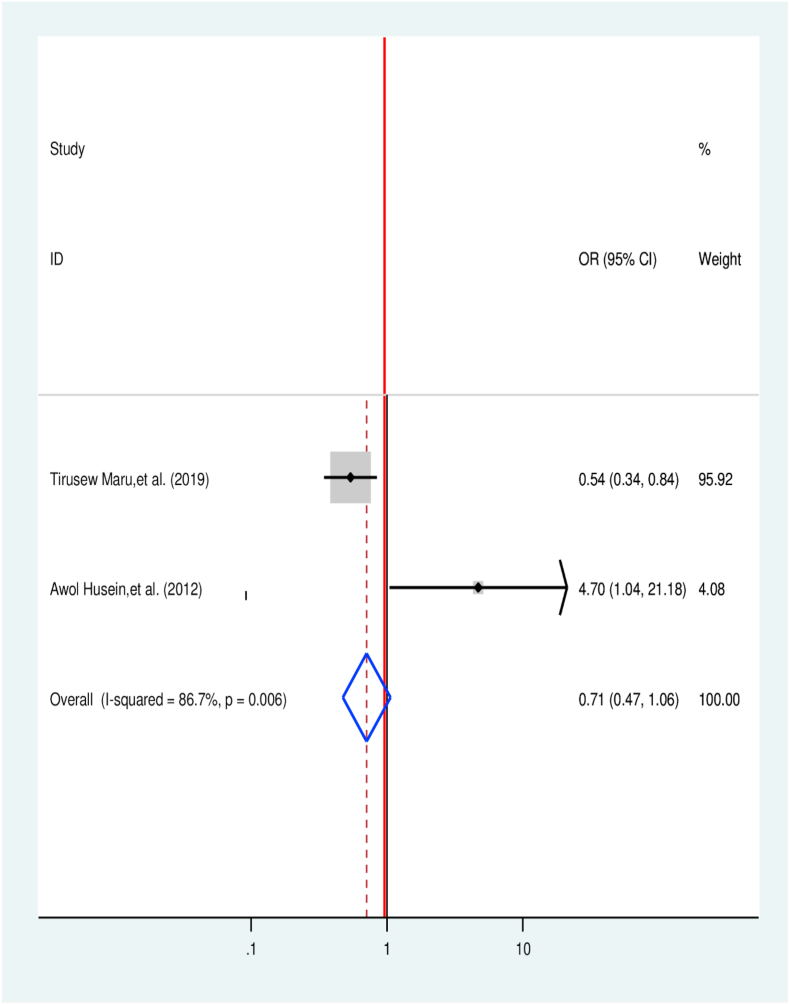
Association between TB susceptibility and TB treatment delay in Ethiopia: Systematic review and Meta-analysis,2024.

Two articles were selected to identify the association between susceptibility toward TB and TB treatment delay [31,46]. Both of the included studies have a significant association, while the overall value reveals that they have no significant association with TB treatment delay (OR = 0.71, 95% CI 0.47, 1.06) (Fig. 8).

**Fig. 9:**
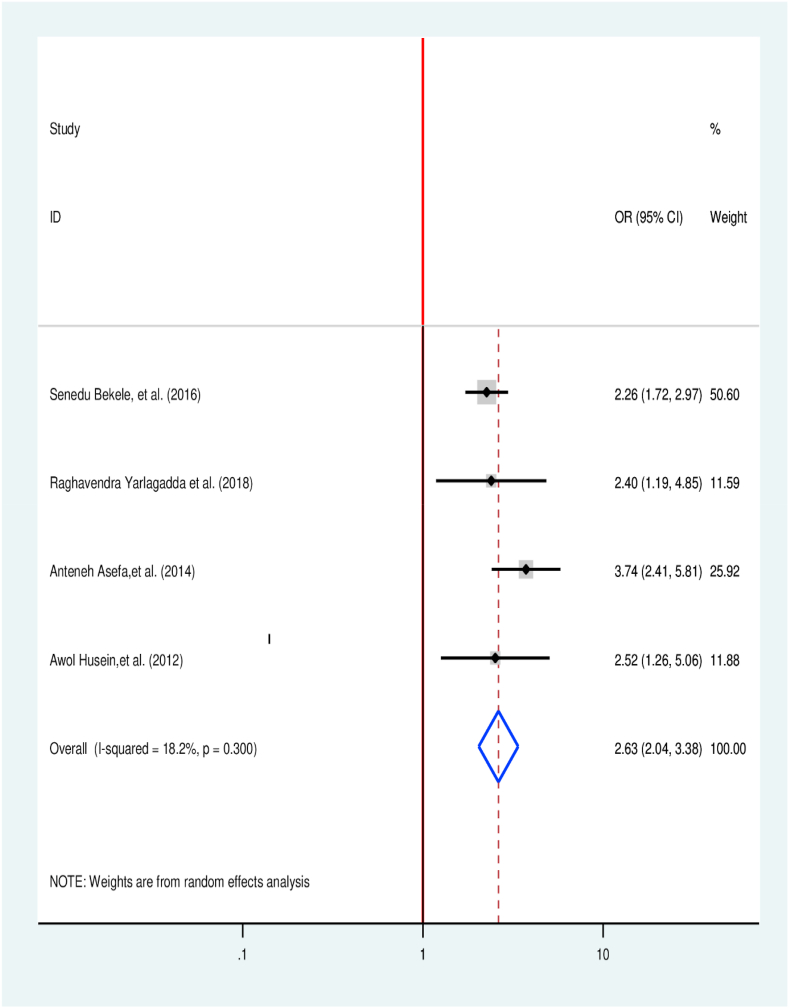
Association between sex of respondent and TB treatment delay in Ethiopia: Systematic review and Meta-analysis,2024.

Four articles were considered to screen the association among sex and treatment delay [31,33,38,42]. All studies have a significant association with treatment delay. Accordingly, female patients were 2.63 higher to delay TB treatment when compared with males (OR = 2.63, 95% CI 2. 04, 3.38)(Fig. 9).

**Fig. 10:**
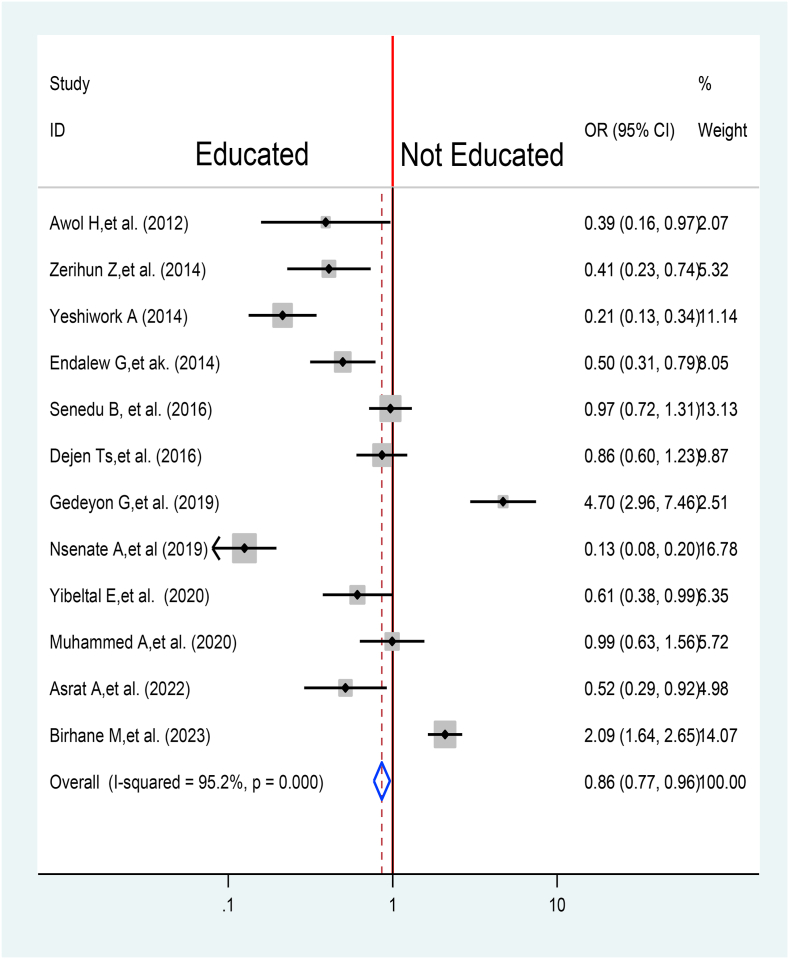
Association between Educational status and TB treatment delay in Ethiopia: Systematic review and Meta-analysis,2024.

A total of 12 articles were pooled together to assess the association between educational status and TB treatment delay [31,[34], [35], [36],^38^,^39^,[47], [48], [49],[51], [52], [53]]. Of twelve studies only three were not significantly associated with TB treatment delay [38,39,49]. However, overall, the pooled effect indicated that educated individuals were 14% less likely to delay their TB treatment when compared with their counter parts (OR = 0.86, 95% CI (0.77, 0.96) (Fig. 10).

**Fig. 11:**
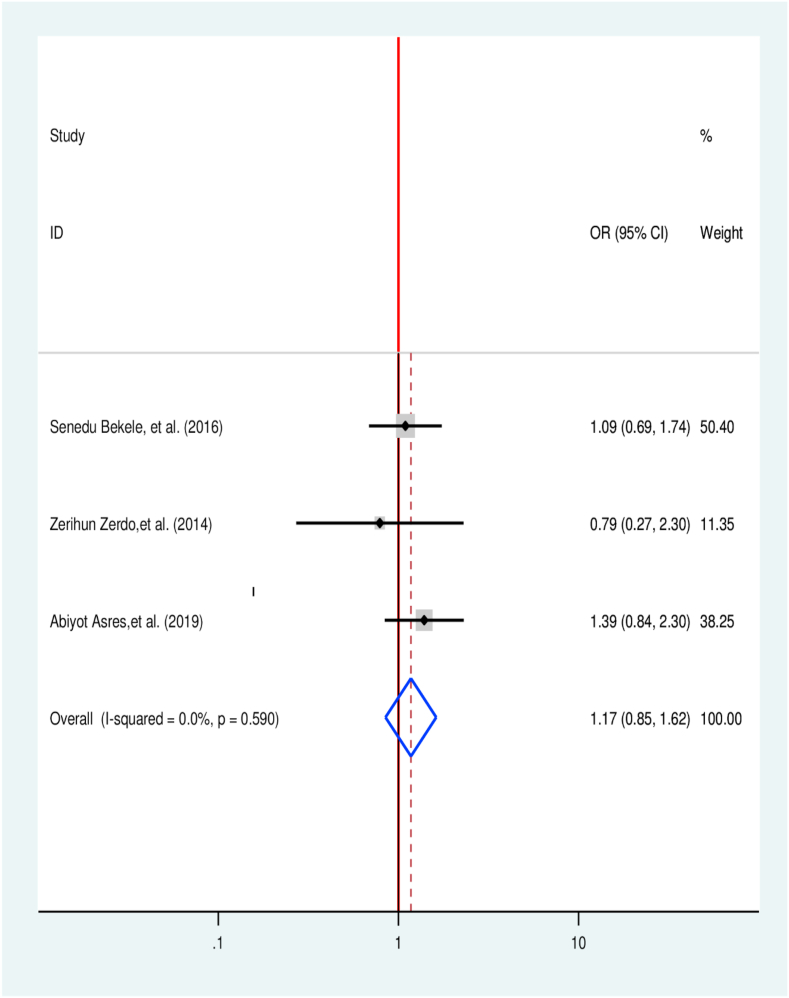
Association between HIV status and TB treatment delay in Ethiopia: Systematic review and Meta-analysis,2024.

Three articles were considered to identify the association among the HIV/AIDS status of patients and treatment delay of TB [34,38,45]. However, the overall effect indicates that there was no significant association between HIV/AIDS and TB treatment delay (Fig. 11).

**Fig. 12:**
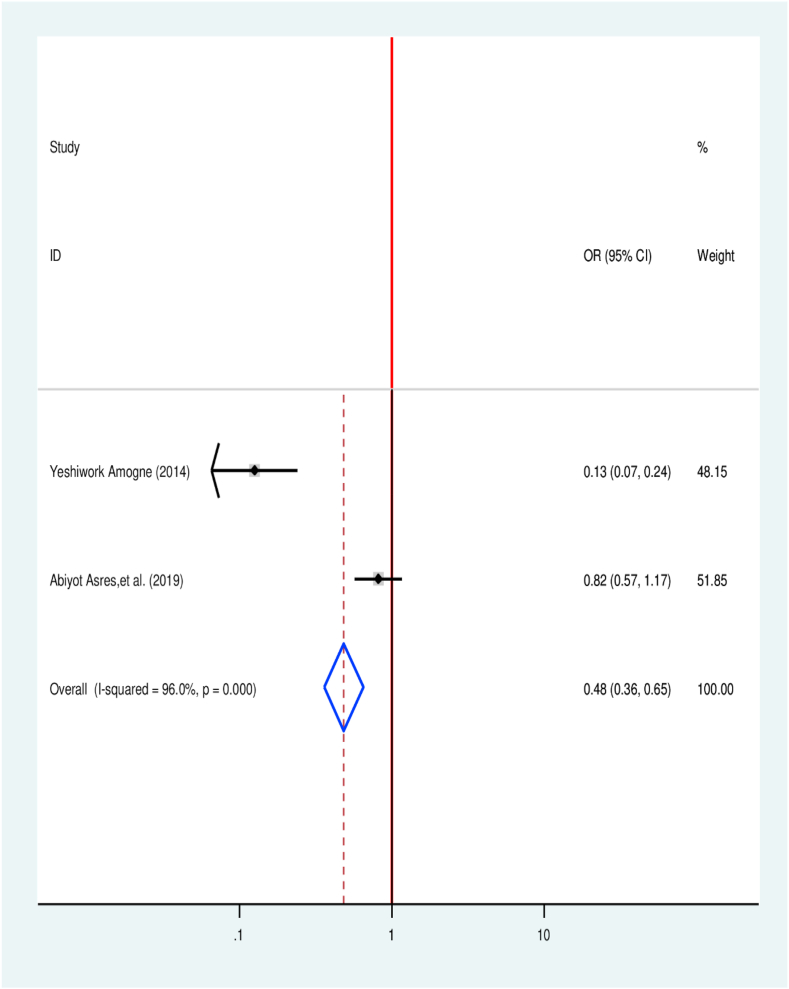
Association between type of TB and TB treatment delay in Ethiopia: Systematic review and Meta-analysis,2024.

Two primary articles were considered to determine the association of TB treatment delay with the type of TB patients experienced currently. One study has no significant association, while one has a significant association. The odds of treatment delay were 52% lower among PTB patients compared to EPTB patients [35,45] (OR = 0.48, 95% CI 0.35, 0.65) (Fig. 12).

**Fig. 13:**
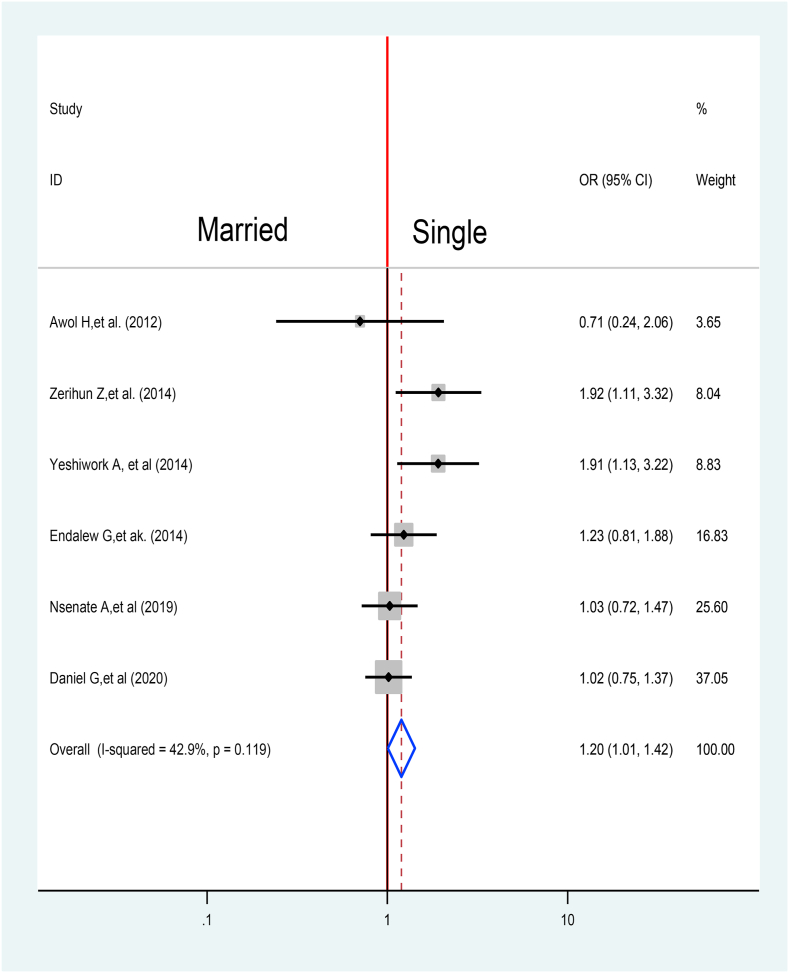
Association between Marital status and TB treatment delay in Ethiopia: Systematic review and Meta-analysis,2024.

The association between marital status and TB Treatment delay was assed using six articles [31,[34], [35], [36],^48^,^50^]. Over all pooled effect reveals that single individuals with TB were 1.2 times at higher risk to delay their treatment when compared with married individuals with TB 1.2 at 95% CI (1.01,1.42) (Fig. 13).

**Fig. 14:**
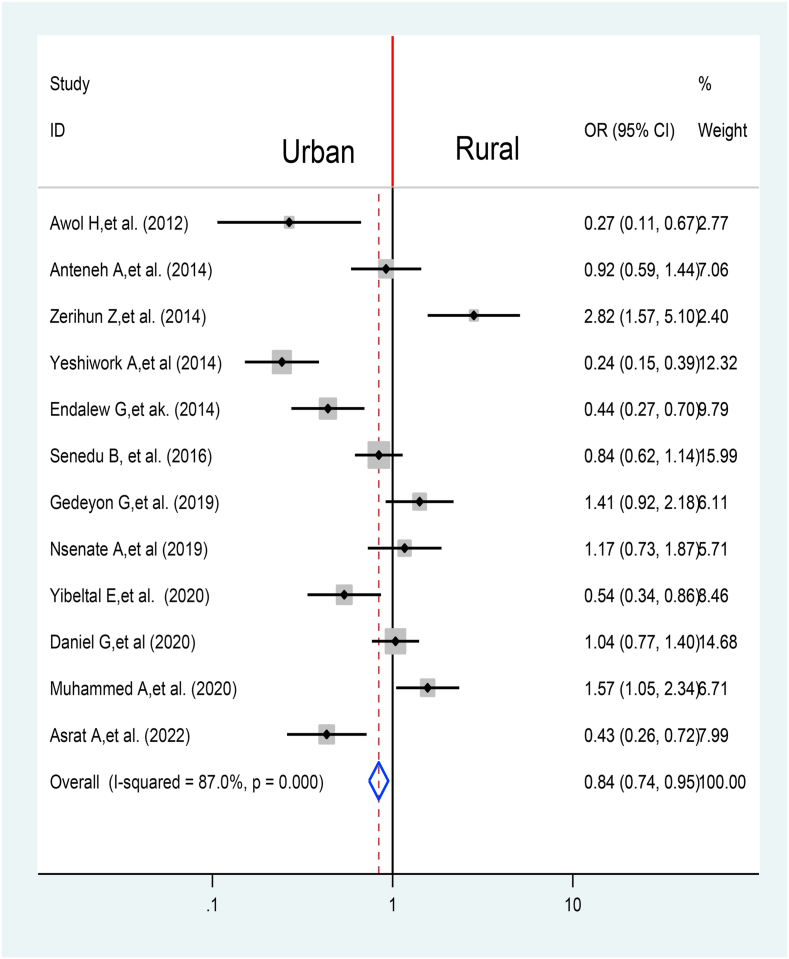
Association between Place of residency and TB treatment delay in Ethiopia: Systematic review and Meta-analysis, 2024.

Twelve articles were used to identify the association between place of residency and TB treatment delay [31,[33], [34], [35], [36],^38^,[47], [48], [49], [50], [51], [52]]. The overall effect indicated that urban residents lower the probability of TB treatment delays by 16% when compared with TB patients residing in rural area 0.84% at 95% CI (0.74%, 0.95%) (Fig. 14).

**Fig. 15:**
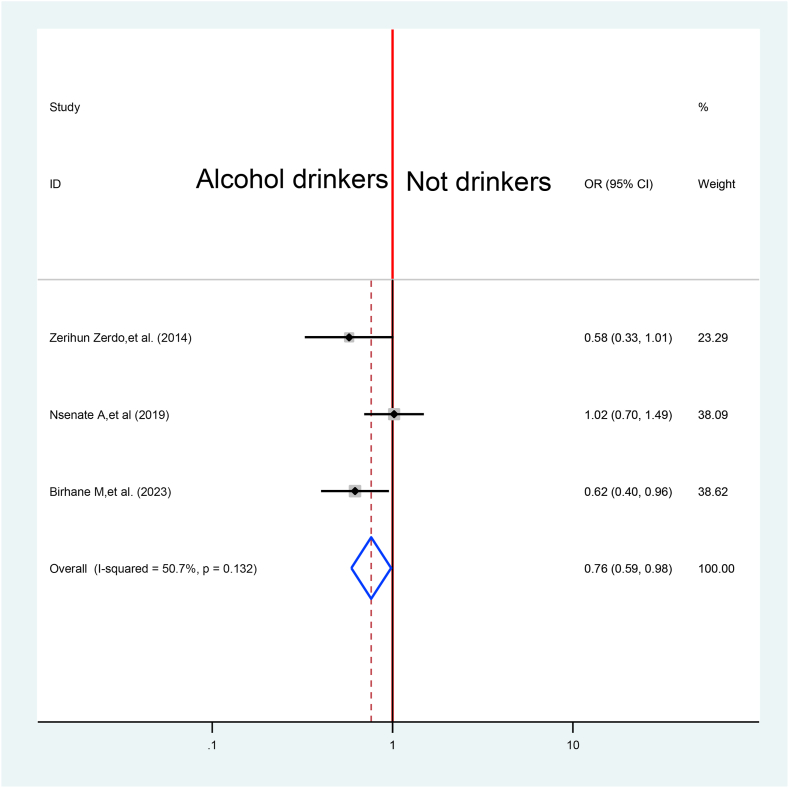
Association between Alcohol drinking and TB treatment delay in Ethiopia: Systematic review and Meta-analysis, 2024.

A total of three studies were pooled to assess the effect of alcohol drinking on TB treatment delay [34,48,53]. The overall effect reveals that alcohol drinkers have probability to delay their treatment with 24% times than non-drinkers 0.76%at 95% CI (0.59%,0.98%) (Fig. 15).

**Fig. 16:**
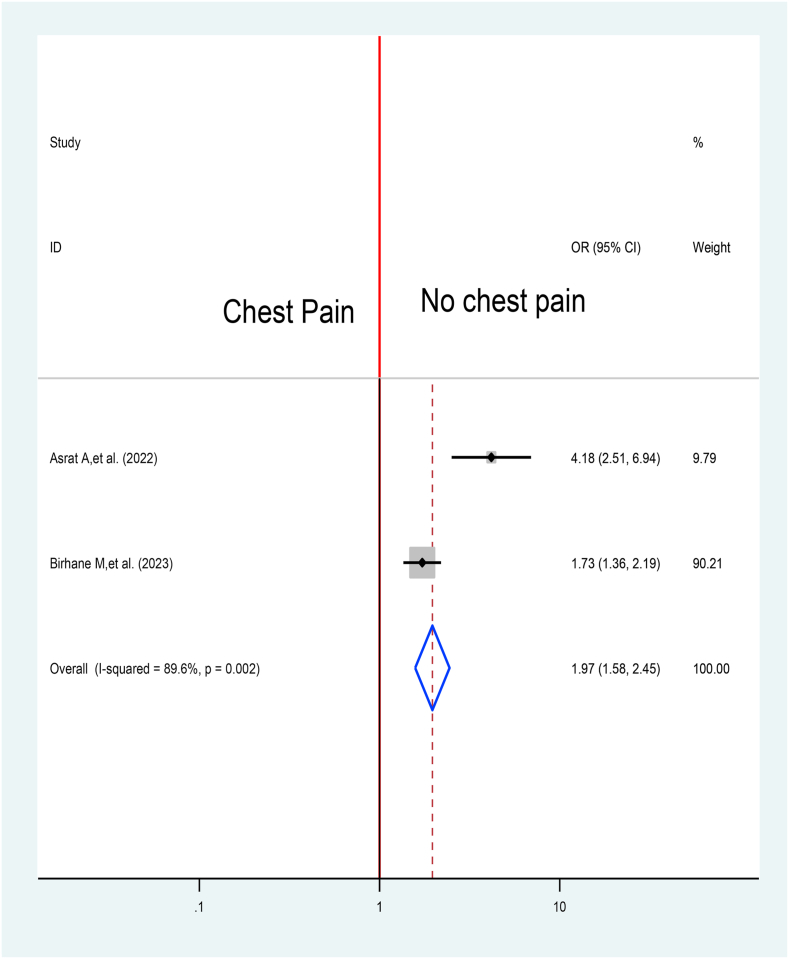
Association between Chest pain and TB treatment delay in Ethiopia: Systematic review and Meta-analysis, 2024.

The association between symptoms like chest pain and TB treatment delay was assessed by pooling results from two articles [52,53]. Accordingly, the overall effect reported that patients with history of no chest pain have around two folds at higher risk to delay their treatment when compare with those who have symptom of chest pain 1.97 at 95% CI (1.58, 2.45) (Fig. 16).

**Fig. 17:**
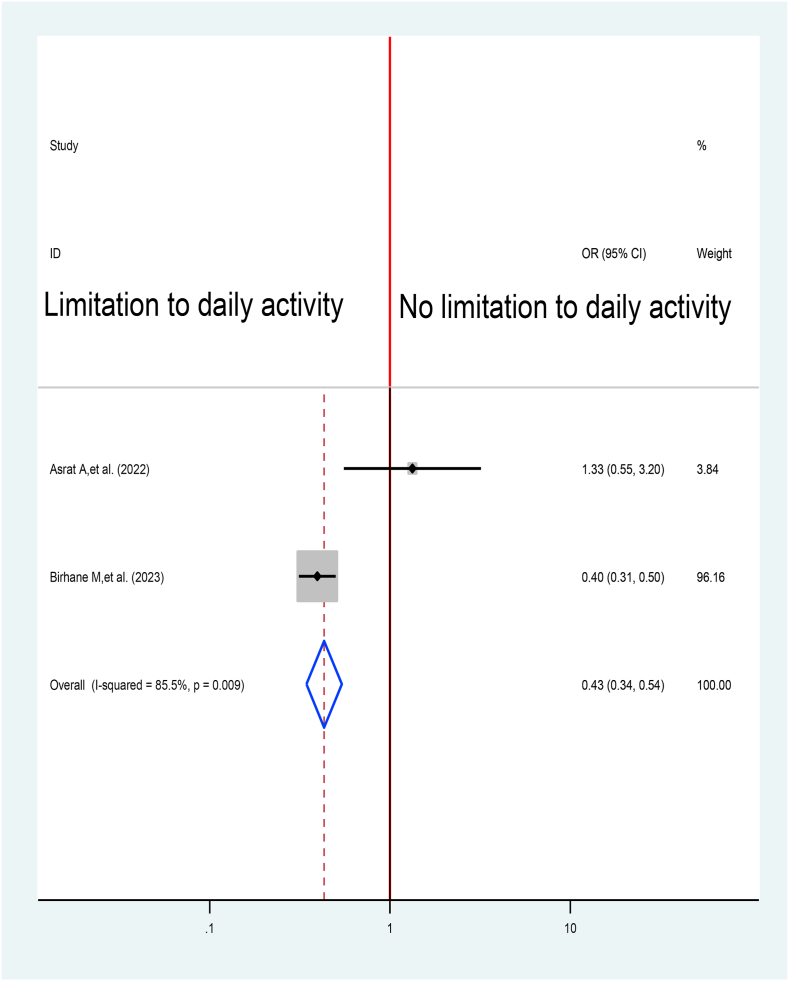
Association between Limitation daily activity and TB treatment delay in Ethiopia: Systematic review and Meta-analysis, 2024.

Tuberculosis disease severity was assessed in relation to TB treatment delay by pooling two evidences [52,53]. Patients those have disease severity that with no limitation on their daily activity have 57% times at lower risk to delay their treatment when compared with those have limitation on their daily activity at 0.43% at 95% CI (0.34%,0.54%) (Fig. 17).

**Fig. 18:**
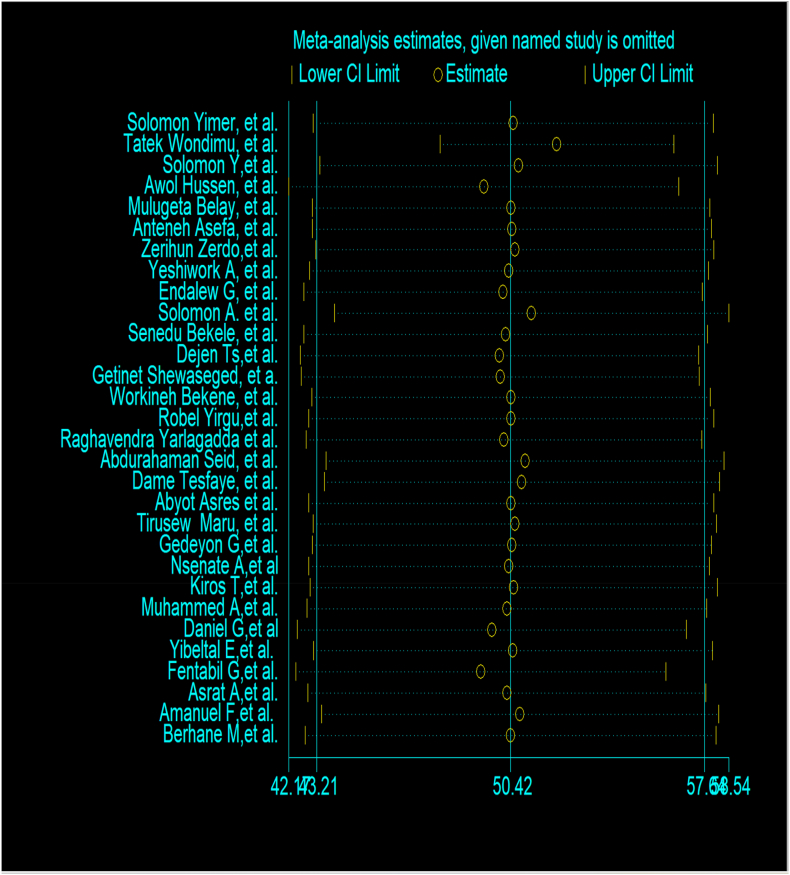
Sensitivity analysis of included studies for TB treatment delay in Ethiopia: Systematic review and Meta-analysis, 2024.

Overall effect of one study over the other was conducted beside the NOS evaluation of individual study. Accordingly, there was no evidence of influence of one study over the other (Fig. 18).

## 4 Discussion

The review indicates as revising the diagnostic and intervention systems importance in Ethiopia, as it was considered high TB is not only preventable but also treatable [1]. Improving current treatment intervention will aid the WHO End-TB Strategy commenced after 2015 [54]. The finding of current analysis reveals that the pooled prevalence of TB treatment delay in Ethiopia 50.42% (43.21%, 57.64%). This result is less than the result from Pakistan at 59.5% [55], and England at 66% [56]. This might be due differences in the health facilities between countries.

Patients who sought services first from governmental health facilities had 20% lower odds to delay TB treatment when compared with patients who sought services first ′from private facilities (OR = 0.80, 95% CI 0.66, 0.96). It in line with results from Pakistan [55] study results from seven countries of the Eastern Mediterranean Region [24] and Suneka of Kenya [57], and Uganda in Wakiso and Mukono district [58]. This is because there is the backward referral of Tb cases after diagnosis in the case of the government facility without cost to nearby health institutions.

Knowing TB lowers 68% of time at lower risk to delay TB treatment in relation to those don't (AOR = 0.32, 95% CI (0.22, 0.46). This can be justified as Poor knowledge about TB will result in treatment delays for TB patients [1]. This is consistent with a systematic review result from the Middle East and North Africa And India [59], Ghana [17], and Uganda [60].

In this study, female with TB were 2.63 times at risk to delay TB treatment in comparison to their counter groups (AOR = 2.63 95% CI (2.04, 3.38). The finding is consistent with study results from Shenzhen, China [61], Nigeria [62], and India [59]. This is due to the reality that tuberculosis is highly prevent among men than females in low, and middle-income countries which might lead to missed attention for early taking women for treatment [63]. In another way, women have financial and physical barriers as well as health literacy that might result to delay in the treatment of tuberculosis [64].

The pooled result indicates that patients having a certain level of education have 14% more likely not to delay treatment in comparison to their counter group (AOR = 0.86, 95% CI 0.77, 0.96). This is similar to the finding from Chad [65] and the results from seven countries of the Eastern Mediterranean Region [24]. This is because educated people are better at seeking treatment earlier than uneducated people.

The pooled effect reveals that patients coming to health facilities from less than or equal to 10 KM were 40% (AOR = 0.60, 95% CI: 0.46, 0.78) less likely to delay treatment when compared with patients coming from greater than 10 KM. It is consistent with a qualitative study conducted in the Somali national regional state [66]result from Afghanistan [67] and finding from seven countries of the Eastern Mediterranean Region [24]. This is because poor access to health care setting will result in a delay in TB treatment, which in turn suggest to the accessibility of health facility for all TB patients is important for appropriate TB control [1].

The pooled result of the current study indicates that patients with pulmonary TB were 52% times less likely to experience TB treatment delay in relation to patients with extrapulmonary (OR = 0.48, 95% CI: 0.35, 0.65). It in line with finding reported from Rwanda in which patients with EPTB and smear-negative pulmonary tuberculosis were at higher risk to delay treatment [68].

In another way, unmarried TB patients were 1.2 more likely to delay treatment when in relation to married TB patients OR = 1.2, 95% CI: 1.01, 1.42). It is consistent with the result from Suneka of Kenya [57]. This can be justified by social support is very important for health-seeking behavior.

Patients those have disease severity that with no limitation on their daily activity have 57% times less likely to delay their treatment when in relation tothose have limitation on their daily activity 0.43% at 95% CI (0.34%,0.54%). This result in line with study by World Health organizations which may be related with living in the suburbs of the study population [8].

Accordingly, the overall effect reported that patients with history of no chest pain have around two folds at higher risk to delay their treatment when compare with those who have symptom of chest pain 1.97 at 95% CI (1.58, 2.45). It inline study result from Uganda [57], and Afghanistan [67]. However, the success of this TB treatment dependent on good TB symptom screening practices to hinder inappropriate delay and irrational use of TB drugs [69]. This implies that if symptoms related with the disease is not reported and treated on time it will contributes for high TB related death. Therefore, clinicians have to give especial attention for TB treatment and detection.

The overall effect indicated that urban residents lower the probability of TB treatment delays by 16% when compared with TB patients residing in rural area 0.84% at 95% CI (0.74%, 0.95%). It is consistent with result from Rwanda [68]. This may be related with inaccessible landscape, the lack of suitable roads, low engagement of other sectors (such as education), professionals a turnover, and a minimum community participation in rural area may be contribute to the problem [70].

The overall effect reveals that alcohol drinkers have probability to delay their treatment with 24% times than non-drinkers 0.76%at 95% CI (0.59%,0.98%). It agrees with evidence undertaken in varies part of the world and alcohol use contributes for global tuberculosis mortality [5].

Further in sub-group analysis indicated that highest magnitude of TB treatment delay was within Somali region. This was related with diagnostic and treatment facilities within the country do not consider pastoralist area of like current area with varying reasons of both dry and wet seasons [66]. In another way such like areas are known of their long distance to health facilities and thus long delay in diagnosis and treatment of tuberculosis [66]. It may also relate with the lack of road facility, limited inter-sectoral integration like (such as education) and an insignificant community engagement, as the cause of most of these obstacles [70].

Furthermore, there might be limitation related with presence of bias within current study and information have to use with caution which might related with sample size and region within which it was undertaken.

## 5 Conclusion and recommendation

Evidence from this systematic review and meta-analysis reported that treatment delay among TB patients was significantly high in Ethiopia. Factors such as knowledge about TB, distance to health facilities less than 10 km, initial contact to a government institution for TB, having some educations, having pulmonary Tuberculosis, urban residency, were protective for treatment delay. Female in gender, no chest pain symptom, disease severity with no restriction on daily activity, alcohol drinkers, and unmarried patients were at higher risk to delay TB treatment. This suggests that clinicians, especially in private clinics providing services like TB in Ethiopia, and policymakers at Ministry of health of Ethiopia should pay attention to these factors to reduce TB treatment delay. Furthermore, Ethiopia have prioritized TB screening and treatment services throughout the country in all setting including private facilities.

## Patient and consent for publication

Not required.

## Ethics approval and consent to participate

Not applicable for current study directly. However, all primary studies included within current review were ethically approved and the research adhered to research ethical principle.

## Consent for publication

Not applicable.

## Data availability statement

All data important to the study were included in the main document.

## Funding

Not applicable.

## CRediT authorship contribution statement

**Getahun Fetensa:** Writing – review & editing, Writing – original draft, Visualization, Validation, Supervision, Software, Resources, Project administration, Methodology, Investigation, Funding acquisition, Formal analysis, Data curation, Conceptualization. **Dessalegn Wirtu:** Validation, Supervision, Resources, Project administration, Methodology, Investigation, Formal analysis, Data curation, Conceptualization. **Belachew Etana:** Writing – review & editing, Writing – original draft, Visualization, Supervision, Funding acquisition, Data curation, Conceptualization. **Bizuneh Wakuma:** Writing – review & editing, Writing – original draft, Visualization, Validation, Supervision, Software, Methodology, Formal analysis, Data curation, Conceptualization. **Tadesse Tolossa:** Writing – original draft, Visualization, Validation, Supervision, Software, Project administration, Methodology, Funding acquisition, Formal analysis, Data curation, Conceptualization. **Jilcha Gugsa:** Writing – review & editing, Writing – original draft, Visualization, Formal analysis, Data curation, Conceptualization. **Dabesa Gobena:** Writing – review & editing, Writing – original draft, Visualization, Supervision, Project administration, Methodology, Formal analysis, Data curation, Conceptualization. **Ginenus Fekadu:** Writing – review & editing, Writing – original draft, Visualization, Supervision, Methodology, Data curation, Conceptualization. **Misganu Teshoma Ragasa:** Writing – review & editing, Writing – original draft, Visualization, Formal analysis, Conceptualization. **Eshetu Ejeta:** Writing – review & editing, Writing – original draft, Visualization, Validation, Supervision, Software, Project administration, Methodology, Formal analysis, Data curation, Conceptualization.

## Declaration of competing interest

The authors declare that they have no known competing financial interests or personal relationships that could have appeared to influence the work reported in this paper.

## References

[1] Vision W. (2017).

[2] Ministry of Health-Ethiopia (2021). Guidelines for clinical and Programmatic management of TB , TB/HIV , DR-TB and Leprosy in Ethiopia.

[3] Place D.S., Lanka S. (2013). WEEKLY epidemiological report A publication of the epidemiology unit Ministry of health. Wkly Epidemiol Rep.

[4] For F., The I., Endtb Strategy (2020). END TB BY 2030.

[5] Kyu H.H., Maddison E.R., Henry N.J., Mumford J.E., Barber R., Shields C. (2018). The global burden of tuberculosis: results from the Global Burden of Disease Study 2015. Lancet Infect. Dis..

[6] Gill C.M., Dolan L., Piggott L.M., McLaughlin A.M. (2022). New developments in tuberculosis diagnosis and treatment. Breathe [Internet].

[7] Tadesse S. (2016). Stigma against tuberculosis patients in Addis Ababa, Ethiopia. PLoS One.

[8] Mediterranean WHORO for the E (2006). http://applications.emro.who.int/dsaf/dsa710.pdf.

[9] Odusanya O.O., Babafemi J.O. (2004). Patterns of delays amongst pulmonary tuberculosis patients in lagos, Nigeria _ BMC public health _ full text. BMC Public Health [Internet].

[10] Christof C., Nußbaumer-Streit B., Gartlehner G. (2020). WHO guidelines on tuberculosis infection prevention and control. Gesundheitswesen.

[11] Cai J., Wang X., Ma A., Wang Q., Han X., Li Y. (2015). Factors associated with patient and provider delays for tuberculosis diagnosis and treatment in Asia: a systematic review and meta-analysis. PLoS One.

[12] Storla D.G., Yimer S., Bjune G.A. (2008). A systematic review of delay in the diagnosis and treatment of tuberculosis. BMC Publ. Health.

[13] Global Economic Impact Of Tuberculosis (2000).

[14] Burden T., Amare D., Deribew A., Deribe K., Dejene T., Tessema G.A. (2018). Tuberculosis burden in Ethiopia from 1990 to 2016 : evidence from the global burden of diseases 2016 study. Ethiop J Heal Sci..

[15] Mohammed H., Oljira L., Roba K.T., Ngadaya E., Ajeme T., Haile T. (2020). Burden of tuberculosis and challenges related to screening and diagnosis in Ethiopia. J Clin Tuberc Other Mycobact Dis [Internet].

[16] Agizew T.B., Dememew Z.G., Leta T., Hiruy N., Tesema E., Abelti E.A. (2022). Prospects for tuberculosis elimination in Ethiopia: feasibility, challenges, and opportunities. Pan Afr Med J..

[17] Osei E., Akweongo P., Binka F. (2015). Factors associated with DELAY in diagnosis among tuberculosis patients in Hohoe Municipality, Ghana. BMC Publ. Health.

[18] Shatil T., Khan N., Yunus F.M., Chowdhury A.S., Reza S., Islam S. (2019). What constitutes health care seeking pathway of TB patients: a qualitative study in rural Bangladesh. J Epidemiol Glob Health.

[19] Abbara A., Collin S.M., Kon O.M., Buell K., Sullivan A., Barrett J. (2019). Time to diagnosis of tuberculosis is greater in older patients : a retrospective cohort review.

[20] Getnet F., Demissie M., Worku A., Gobena T., Tschopp R., Girmachew M. (2019). Delay in diagnosis of pulmonary tuberculosis increases the risk of pulmonary cavitation in pastoralist setting of Ethiopia. BMC Pulm. Med..

[21] Wondimu T., W/Michael K., Kassahun W., Getachew S. (2007). Delay in initiating tuberculosis treatment and factors associated among pulmonary tuberculosis patients in East Wollega, Western Ethiopia. Ethiop. J. Health Dev..

[22] Hussen A., Biadgilign S., Tessema F., Mohammed S., Deribe K., Deribew A. (2012). Treatment delay among pulmonary tuberculosis patients in pastoralist communities in Bale Zone, Southeast Ethiopia. BMC Res. Notes.

[23] Moher D., Liberati A., Tetzlaff J., Altman D.G., Antes G., Atkins D. (2009). Preferred reporting items for systematic reviews and meta-analyses: the PRISMA statement. PLoS Med..

[24] Bassili A., Seita A., Baghdadi S., Alabsi A., Abdilai I., Agboatwalla M. (2008). Diagnostic and treatment delay in tuberculosis in 7 countries of the Eastern Mediterranean Region. Infect. Dis. Clin. Pract..

[25] Fanta A., Daniel A. (2023). Nursing and health care delay in healthcare-seeking behavior and its associated factors among tuberculosis patients attending TB clinic in hawassa city health facilities in Sidamma region. Hawassa. Int Arch Nurs Heal Care..

[26] Tedla K., Medhin G., Berhe G., Mulugeta A., Berhe N. (2020). Delay in treatment initiation and its association with clinical severity and infectiousness among new adult pulmonary tuberculosis patients in Tigray , northern. BMC Infect. Dis..

[27] Adenager G.S., Alemseged F., Asefa H., Gebremedhin A.T. (2017). Factors associated with treatment delay among pulmonary tuberculosis patients in public and private health facilities in Addis Ababa, Ethiopia. Tuberc Res Treat..

[28] Getnet F., Demissie M., Worku A., Gobena T., Tschopp R., Seyoum B. (2020). Longer delays in diagnosis and treatment of pulmonary tuberculosis in pastoralist setting , eastern Ethiopia. Risk Manag. Healthc. Pol..

[29] Yimer S., Bjune G., Alene G. (2005). Diagnostic and treatment delay among pulmonary tuberculosis patients in Ethiopia: a cross sectional study. BMC Infect. Dis..

[30] Yimer S.A., Hansen C.H., Bjune G.A. (2011). The perspective of private practitioners regarding tuberculosis case detection and treatment delay in Amhara Region, Ethiopia: a cross-sectional study. BMC Res Notes [Internet].

[31] Hussen A., Biadgilign S., Tessema F., Mohammed S., Deribe K., Deribew A. (2012). Treatment delay among pulmonary tuberculosis patients in pastoralist communities in Bale Zone, Southeast Ethiopia. BMC Res Notes [Internet].

[32] Belay M., Bjune G., Ameni G., Abebe F. (2012). Diagnostic and treatment delay among Tuberculosis patients in Afar Region, Ethiopia: a cross-sectional study. BMC Publ. Health.

[33] Asefa A., Teshome W. (2014). Total delay in treatment among smear positive pulmonary tuberculosis patients in five primary health centers, Southern Ethiopia: a cross sectional study. PLoS One.

[34] Zeleke Z.Z. (2014). Treatment delay among smear positive pulmonary tuberculosis patients in south Ethiopia: a cross-sectional study. Sci. J. Publ. Health.

[35] Mekonnen Y.A. (2014). Delay for first consultation and associated factors among tuberculosis patients in bahir dar town administration, North west Ethiopia. Am. J. Health Res..

[36] Gebeyehu E., Azage M., Abeje G. (2014). Factors associated with patient ’ s delay in tuberculosis treatment in bahir factors associated with patient ’ s delay in tuberculosis treatment in bahir dar city administration , northwest Ethiopia. Hindawi Publ. Corp. BioMed. Res. Int..

[37] Yimer S.A., Norheim G., Namouchi A., Zegeye E.D., Kinander W., Tønjum T. (2015). Mycobacterium tuberculosis lineage 7 strains are associated with prolonged patient delay in seeking treatment for pulmonary tuberculosis in Amhara region, Ethiopia. J. Clin. Microbiol..

[38] Gebreegziabher S.B., Bjune G.A., Yimer S.A. (2016). Patients' and health system's delays in the diagnosis and treatment of new pulmonary tuberculosis patients in West Gojjam Zone, Northwest Ethiopia: a cross-sectional study. BMC Infect Dis [Internet].

[39] Tsegaye D., Abiy E., Mesele T., Tadesse T. (2016). Delay in seeking health care and associated factors among pulmonary iMedPub journals delay in seeking health care and associated factors among pulmonary tuberculosis patients in North wollo zone , northeast Ethiopia : institution based cross-sectional stu. Arch. Clin. Microbiol..

[40] Bekana W., Sisay M., Baye Y. (2017). Evaluation of factors affecting patient delay in the diagnosis and treatment of TB among TB patients attending in hiwot fana specialized university hospital, harar, eastern Ethiopia. J Anc Dis Prev Remedies.

[41] Yirgu R., Lemessa F., Hirpa S., Alemayehu A., Klinkenberg E. (2017). Determinants of delayed care seeking for TB suggestive symptoms in Seru district, Oromiya region, Ethiopia: a community based unmatched case-control study. BMC Infect. Dis..

[42] Mulugeta R.Y.T., Gashe F., Tatiparthi R. (2018). Delay for treatment and associated factors among tuberculosis patients in jimma town, Ethiopia. Int. J. Pharm. Pharmaceut. Res..

[43] Seid A., Metaferia Y. (2018). Factors associated with treatment delay among newly diagnosed tuberculosis patients in Dessie city and surroundings, Northern Central Ethiopia: a cross-sectional study. BMC Publ. Health.

[44] Tesfaye D., Dubale S. (2018). Current knowledge of TB patients on initiation of treatment in mettu town. Biomed J Sci Tech Res.

[45] Asres A., Jerene D., Deressa W. (2019). Delays to anti-tuberculosis treatment intiation among cases on directly observed treatment short course in districts of southwestern Ethiopia: a cross sectional study. BMC Infect. Dis..

[46] Wondawek T.M., Ali M.M. (2019). Delay in treatment seeking and associated factors among suspected pulmonary tuberculosis patients in public health facilities of Adama town, eastern Ethiopia. BMC Publ. Health.

[47] Getahun G., Beyene T., Abebe L. (2019). Journal of drug delivery and therapeutics tuberculosis ’ total health care system and patient side treatment delay and associated factors among pulmonary tuberculosis patients at hadiya. J. Drug Deliv. Therapeut..

[48] Awoke N., Dulo B., Wudneh F. (2019). Total delay in treatment of tuberculosis and associated factors among new pulmonary TB patients in selected health facilities of gedeo zone , southern Ethiopia , 2017/18. Hindawi Interdiscip Perspect Infect Dis..

[49] Abdu M., Balchut A., Girma E., Mebratu W. (2020). Patient delay in initiating tuberculosis treatment and associated factors in Oromia special zone , Amhara region. Hindawi Pulm Med.

[50] Datiko D.G., Jerene D., Suarez P. (2020). Patient and health system delay among TB patients in Ethiopia : nationwide mixed method cross-sectional study. BMC Publ. Health.

[51] Yeafaywabmb Gobeza (2020). Delay for tuberculosis treatment and its predictors among adult tuberculosis patients at debremarkos town public health. Hindawi Tuberc Res Treat..

[52] Arja A., Bogale B., Gebremedhin M. (2022). Health system delay and its associated factors among tuberculosis patients in Gamo Zone public health facilities. J Clin Tuberc Other Mycobact Dis [Internet].

[53] Megerssa B., Id E., Sagbakken M., Gradmann C. (2023). Total delay and associated factors among tuberculosis patients in Jimma Zone , Southwest Ethiopia. PLoS One [Internet].

[54] Sulis G., Centis R., Sotgiu G., D'Ambrosio L., Pontali E., Spanevello A. (2016). Recent developments in the diagnosis and management of tuberculosis. npj Prim Care Respir Med.

[55] Saqib S.E., Ahmad M.M., Amezcua-Prieto C., Virginia M.R. (2018). Treatment delay among pulmonary tuberculosis patients within the Pakistan national tuberculosis control program. Am. J. Trop. Med. Hyg..

[56] Loutet M.G., Sinclair C., Whitehead N., Cosgrove C., Lalor M.K., Thomas H.L. (2018). Delay from symptom onset to treatment start among tuberculosis patients in England, 2012-2015. Epidemiol. Infect..

[57] Patients T., Nyatichi F.O., Amimo F.A., Nabie B., Ondimu T.O. (2016). Factors contributing to delay in seeking treatment among pulmonary health education research & development.

[58] Buregyeya E., Criel B., Nuwaha F., Colebunders R. (2014). Delays in diagnosis and treatment of pulmonary tuberculosis in Wakiso and Mukono districts, Uganda. BMC Publ. Health.

[59] Konda S.G., Giri P. (2020). A Cross-Sectional Study Determinants of Delays in Diagnosis and Treatment of Pulmonary Tuberculosis in a New Urban Township in India.

[60] Sekandi J.N., Zalwango S., Martinez L., Handel A., Kakaire R., Nkwata A.K. (2015). Four degrees of separation: social contacts and health providers influence the steps to final diagnosis of active tuberculosis patients in urban Uganda. BMC Infect. Dis..

[61] Xu X., Liu J.H., Cao S.Y., Zhao Y., Dong X.X., Liang Y. (2013). Delays in care seeking, diagnosis and treatment among pulmonary tuberculosis patients in Shenzhen, China. Int. J. Tubercul. Lung Dis..

[62] Ukwaja K.N., Alobu I., Nweke C.O., Onyenwe E.C. (2013). Healthcare-seeking behavior , treatment delays and its determinants among pulmonary tuberculosis patients in rural Nigeria. a cross- sectional study.

[63] Horton K.C., MacPherson P., Houben R.M.G.J., White R.G., Corbett E.L. (2016). Sex differences in tuberculosis burden and notifications in low- and middle-income countries: a systematic review and meta-analysis. PLoS Med..

[64] Yang W.T., Gounder C.R., Akande T., De Neve J.W., McIntire K.N., Chandrasekhar A. (2014). Barriers and delays in tuberculosis diagnosis and treatment services: does gender matter?. Tuberc Res Treat..

[65] Ngangro N.N., Ngarhounoum D., Ngangro M.N., Rangar N., Siriwardana M.G., Des Fontaines V.H. (2012). Pulmonary tuberculosis diagnostic delays in Chad: a multicenter, hospital-based survey in Ndjamena and Moundou. BMC Publ. Health.

[66] Gele A.A., Sagbakken M., Abebe F., Bjune G.A. (2010). Barriers to tuberculosis care: a qualitative study among Somali pastoralists in Ethiopia. BMC Res. Notes.

[67] Sabawoon W., Sato H., Kobayashi Y. (2012). Delay in the treatment of pulmonary tuberculosis: a report from Afghanistan. Environ. Health Prev. Med..

[68] Lorent N., Mugwaneza P., Mugabekazi J., Gasana M., Van Bastelaere S., Clerinx J. (2008). Risk factors for delay in the diagnosis and treatment of tuberculosis at a referral hospital in Rwanda. Int. J. Tubercul. Lung Dis..

[69] World Health Organization (2020).

[70] Ereso B.M., Yimer S.A., Gradmann C., Sagbakken M. (2020). Barriers for tuberculosis case finding in Southwest Ethiopia: a qualitative study. PLoS One.

